# Physiological and Psychological Relaxation Effects of Fir Essential Oil on University Students

**DOI:** 10.3390/ijerph19095063

**Published:** 2022-04-21

**Authors:** Choyun Kim, Chorong Song

**Affiliations:** Department of Forest Science, Kongju National University, 54 Daehak-ro, Yesan-eup, Yesan-gun 32439, Korea; kcy2605@gmail.com

**Keywords:** forest healing, forest therapy, stress relief, relaxation, fir essential oil, sex comparison, heart rate variability, heart rate, profile of mood states, state-trait anxiety inventory

## Abstract

Numerous studies have reported a significant increase in stress experienced by students owing to the COVID-19 pandemic. Recently, interest in stress management using nature-derived substances has increased. However, studies examining the effects of olfactory stimulation by fir are lacking. The aim of this study was to investigate the physiological and psychological effects of inhaling fir essential oil. Additionally, differences between male and female participants were compared. Twenty-six university students (16 female and 10 male students; mean age, 21.5 ± 1.9 years) participated in this study. Fir essential oil was used for olfactory stimulation, with normal room air as the control. The odor was administered for 3 min. Heart rate variability and heart rate were used as indicators of autonomic nervous system activity. The Profile of Mood States and State-Trait Anxiety Inventory were used as psychological indicators. The ln(Low Frequency/High Frequency) ratio, which is an indicator of sympathetic nervous activity reflecting a stressful or aroused state during stimulation with fir essential oil, was significantly lower than during the control condition. Assessment of psychological indicators showed that the positive mood of “vigor” improved significantly and negative moods of “tension–anxiety”, “depression”, “anger–hostility”, “fatigue” and anxiety levels reduced significantly after inhaling fir essential oil compared to the control condition. This study showed that inhalation of fir essential oil has physiologically and psychologically relaxing effects, with differences in results depending on the sex of the participants.

## 1. Introduction

In December 2019, the world plunged into chaos when a novel strain of coronavirus was discovered. As the number of confirmed cases continued to increase, the WHO declared COVID-19 a Public Health Emergency of International Concern on 30 January 2020, and a pandemic on 11 March 2020. This was the third pandemic in the history of the world. To prevent the spread of COVID-19, all countries chose to restrict face-to-face contact and movement through measures such a social distancing, immigration control, securing a period of self-isolation, and blocking specific areas.

Students were prohibited from face-to-face learning because of the increased risk of infection. To replace in-person teaching, most classes were conducted in digital and online formats. These rapid changes in the educational environment and lifestyle have had a negative impact on students [1,2,3,4,5]. The European Union revealed that approximately 60% of students in France, Spain, and Poland experienced increased stress and anxiety due to the COVID-19 pandemic [1,2,3] and 38% of students in Turkey reported being anxious about the infection [4]. More than half of the students in the United Kingdom felt lonely every day because of the continued COVID-19 pandemic and restrictive measures such as social distancing [6]. A qualitative study conducted in Korea to determine why university students were stressed during COVID-19 found that there were many concerns about poor academic quality, disconnection from social relations, job loss, and anxiety about health due to the COVID-19 pandemic [5].

If negative emotions persist and are not properly managed, they can have negative effects on both physical and mental health. Physical symptoms include headaches, increased blood pressure, and hives, whereas psychological symptoms comprise anxiety, depression, loss of motivation, and helplessness [7]. In addition, excessive stress can decrease quality of life, happiness, and life satisfaction [8]. Therefore, it is important to manage stressful states.

Because humans have spent most of their history in forests, they have evolved to adapt to forest environments [9]. However, due to rapid industrialization and urbanization in the late 19th century, their residential sites moved from forests to cities. The difference between forest and urban environments can often lead to stress in people [10]. Therefore, humans feel comfortable when visiting forests [11,12,13] and contacting with various resources derived from forests [14,15,16,17]. “Forest healing” is an attempt to induce stress relief, mental and physical relaxation, and link health promotion with immunity improvement by utilizing these mechanisms. It is also called “forest therapy,” “forest bathing,” or “shinrin-yoku.”

Various studies have revealed the benefits of engaging in activities in forest environments. Forest-viewing and -walking can lower concentrations of cortisol, pulse rate, blood pressure, and cerebral activity in the areas of the prefrontal cortex, thus increasing parasympathetic nervous activity and decreasing sympathetic nervous activity [11,12]. Meditation and walking programs in the forest decreased systolic and diastolic blood pressure, urinary adrenaline, and serum cortisol levels [13]. Psychologically, it was reported that natural and relaxed mood increased and negative mood decreased.

However, it is difficult to visit the forest environment directly due to movement constraints due to the COVID-19 pandemic and time constraints due to busy schedules. Accordingly, interest in forest healing resources that can indirectly affect healing without visiting the forest is increasing. Among the various factors of forests, odor has been used to promote mental and physical health in the past [14].

Many studies have been performed on cypress that emits a large amount of phytoncide. Studies using leaf-extracted oil, wood-extracted oil, and wood chips have shown positive effects on physiological and psychological relaxation and the improvement of immune function [15,16,17]. However, verification of the effectiveness of other tree species is insufficient.

In Korea, cypress is limited to the southern region owing to its ecological characteristics. Therefore, since the properties are similar, attention should be paid to the species belonging to the northern part of the central temperate region. Previous studies have shown that fir trees have higher concentrations of trans-caryophyllene and bornyl acetate than other tree species [18]. Trans-caryophyllene and bornyl acetate are terpene compounds in trees; trans-caryophyllene has anti-inflammatory effects [19] and bornyl acetate has calming and relaxing effects [20].

The fir (*Abies holophyla*) is an evergreen coniferous species belonging to the pine tree family found in the northern central part of Korea. Fir trees contain various substances that are known to be effective in treating the symptoms of cold, abdominal pain, indigestion, rheumatic diseases, and vascular and lung diseases, and are widely used as folk remedies [21]. Hong et al. [22] demonstrated that inhaling fir oil positively affects stress, sleep quality, and fatigue in middle-aged women. Baik et al. [23] examined physiological responses when inhaling fir essential oil in office workers with a small sample size. They reported that the sympathetic nervous activity, heart rate, and blood pressure decreased significantly, whereas the parasympathetic nervous activity increased. However, there is a lack of research that comprehensively examines the physiological and psychological responses when fir essential oil is inhaled. Accordingly, this study aimed to investigate the physiological and psychological effects of the inhalation of fir essential oil.

In addition, most studies on olfactory stimulation of forest-derived substances reported effects in female [15,16,17], and there is a lack of research on the physiological effects in males, on which more data are needed. This study also examined the differences in the physiological and psychological effects of fir essential oil inhalation between male and female university students.

## 2. Materials and Methods

### 2.1. Participants

Twenty-six university students were recruited for this study. A notice of participant recruitment was posted on the school bulletin board of Kongju National University. Individuals treated for any disease, or those with anosmia, parosmia, or physical and psychological issues were excluded from the present study. To avoid distortions or biases, students from various majors were recruited. Therefore, 16 female and 10 male university students participated in this experiment (mean age, 21.5 ± 1.9 years). Detailed information about the participants was provided in Table 1. This study was approved by the Institutional Review Board of Kongju National University (IRB number: KNU_IRB_2021-35). All participants were informed of the aim and procedures involved in the experiment and they provided written informed consent to participate.

### 2.2. Olfactory Stimulation

Fir essential oil extracted by steam distillation using fir leaves native to India (Kanta Enterprises Pvt. Ltd., New Delhi, India) was used for olfactory stimulation in the experimental condition. Room air was used in the control condition. A digital diffuser was used for olfactory stimulation and was fixed at 3 m from the participant’s position. The machine uses the compression atomization method, which involves spraying the essential oil by compressing it into fine particles. A total of 38 μL of fir essential oil was emitted into the experiment room for 1 min. The size of the experiment room was 22.68 m^3^. Participants could comfortably inhale with their eyes closed.

### 2.3. Experimental Design

**Figure 1:**
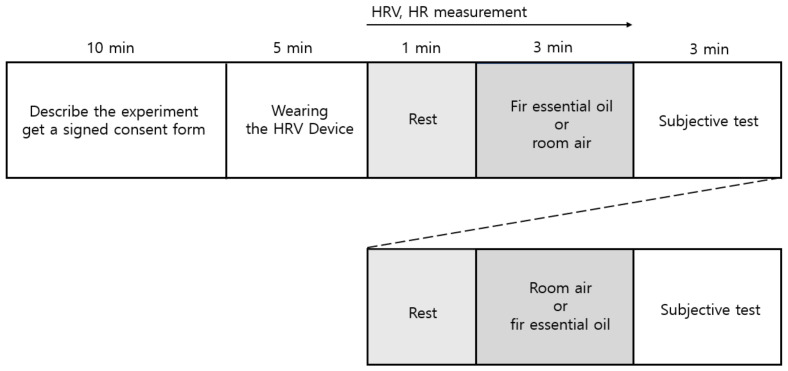
Experimental protocol.

The experimental procedure is illustrated in Figure 1. Upon arriving in the experiment room, participants voluntarily completed the consent form after hearing the purpose and procedure of the experiment and before starting the experiments. After the instrument for measuring physiological responses was attached, participants sat in a chair for 5 min. Participants closed their eyes and rested for 1 min, followed by exposure to fir essential oil or room air for 3 min. After olfactory stimulation, participants opened their eyes and conducted a subjective evaluation for 3 min. This study adopted a within-subject experimental design. Physiological and psychological responses were compared when the same participant inhaled fir essential oil or room air. To eliminate the influence of order, approximately half of the participants were administered stimulation in the following order: exposure to fir essential oil, followed by room air (control). The remaining participants were administered the control, followed by the fir essential oil.

### 2.4. Physiological Measurement

Heart rate variability (HRV) and heart rate, which were used to quantify autonomic nervous activity, were measured using a wearable electrocardiogram sensing system (myBeat; Union Tool Co., Tokyo, Japan). Frequency spectra were generated using an HRV software tool (MemCalc/Win; GMS, Tokyo, Japan). HRV was analyzed for the periods between consecutive R waves (R-R intervals). In this study, two broad HRV spectral components were calculated: low frequency (LF; 0.04–0.15 Hz) and high frequency (HF; 0.15–0.40 Hz). The HF power reflected parasympathetic nervous activity. The LF/HF ratio reflected sympathetic nervous activity. The HF power and LF/HF ratio were transformed to their natural logarithmic values, ln(HF) and ln(LF/HF), respectively (Kobayashi, 2012). The means of the data were acquired for 60 s of rest and during 180 s of order administration. Heart rate was calculated as the number of heart beats per minute.

### 2.5. Psychological Measurement

To subjectively evaluate the psychological effects of olfactory stimulation, participants answered the following two questionnaires after the experimental and control conditions.

#### 2.5.1. Profile of Mood State (POMS)

Participants’ mood states were evaluated using the Korean shortened mood scale (K-POMS-B). It consists of 30 questions evaluated on a five-point Likert scale. POMS scores were determined for the following six subscales: “tension–anxiety (T–A)”, “depression (D)”, anger–hostility (A–H)”, “fatigue (F)”, “confusion (C)”, and “vigor (V).” The total mood disturbance (TMD) score was calculated using the formula: [(T–A) + (D) + (A–H) + (F) + (C) − (V)].

#### 2.5.2. State-Trait Anxiety Inventory (STAI)

STAI-X-1 was used to assess participants’ state anxiety levels. It comprises 20 questions that evaluate “how you are feeling now?”. Each question is assessed on a four-point Likert scale, with higher scores indicating higher levels of anxiety.

### 2.6. Data Analysis

Six participants were excluded because of errors in HRV data collection. All statistical analyses were performed using SPSS 26.0 (IBM Corp., Armonk, NY, USA). We used a paired sample *t*-test to compare physiological responses between the experimental and control conditions. The G-power 3.1 program was used to calculate the number of statistically valid participants. The setting values were as follows: effect size, 0.80; significance level, 0.05; power, 0.95; analysis method, paired *t*-test; difference between two dependent means (matched pairs). As a result, the minimum number of samples was calculated to be 19. The number of samples in this study was 20; therefore, the difference was statistically significant. Wilcoxon signed-rank test was used to analyze differences in the psychological indices. This study used a one-sided test because we hypothesized that participants would relax by inhaling fir essential oil.

## 3. Results and Discussion

### 3.1. All Participants

#### 3.1.1. Physiological Measurement

**Figure 2:**
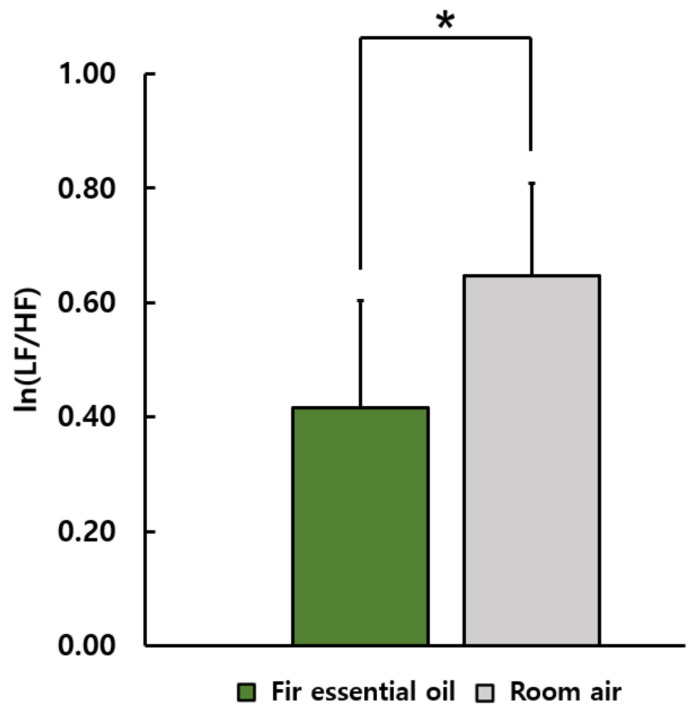
The ln(LF/HF) values of heart rate variability during the inhaling fir essential oil and room air. *N* = 20, mean ± standard error, * *p* < 0.05 by paired *t*-test (one-sided).

Figure 2 shows the ln(LF/HF) ratio, an indicator of sympathetic nervous activity in HRV, during inhalation of fir essential oil and room air. The ln(LF/HF) ratio during stimulation using fir essential oil was significantly lower than during the control condition (fir essential oil: 0.42 ± 0.19, room air: 0.65 ± 0.16; *p* < 0.05). However, no significant differences were found in ln(HF) and heart rate.

These results are consistent with previous studies [15,24]. Research on the effect of inhalation of cedrol, a major compound found in Japanese cedar, has demonstrated that during inhalation of cedrol, sympathetic nervous activity, heart rate, and blood pressure were reduced and parasympathetic nervous activity was induced [24]. Inhalation of α-pinene and D-limonene, major compounds found in *Cryptomeria japonica* and *Pinus densiflora*, induced parasympathetic nervous activity and reduced heart rate [16,25]. In addition, inhalation of cypress leaf oil increased parasympathetic nervous activity and decreased oxy-Hb concentration in the right prefrontal cortex [15]. These results suggest that natural derived elements relaxed autonomic nervous activity.

#### 3.1.2. Physiological Measurement

**Figure 3:**
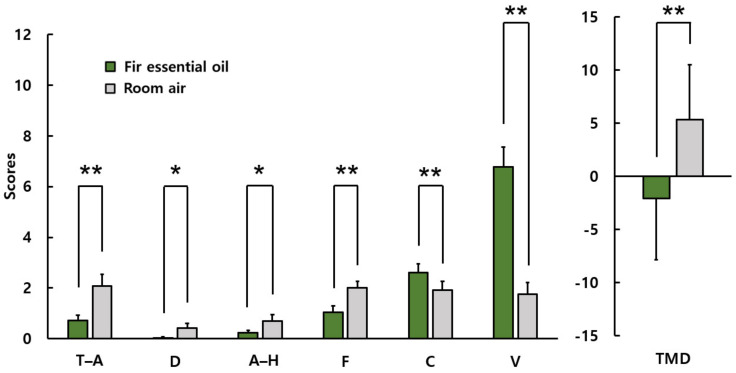
POMS scores after inhaling fir essential oil and room air. T–A: tension–anxiety; D: depression; A–H: anger–hostility; F: fatigue; C: confusion; V: vigor; TMD: Total Mood Disturbance *N* = 26, mean ± standard deviation, ** *p* < 0.01, * *p* < 0.05 by Wilcoxon singed-rank test (one-sided).

Figure 3 shows the POMS results. The subscale scores when inhaling fir essential oil and room air were as follows: tension and anxiety, 0.7 ± 1.0 vs. 2.1 ± 2.3; depression, 0.0 ± 0.2 vs. 0.4 ± 0.9; anger–hostility, 0.2 ± 0.5 vs. 0.7 ± 1.3; fatigue, 1.0 ± 1.3 vs. 2.0 ± 1.4; confusion, 2.6 ± 1.7 vs. 1.9 ± 1.8; vigor, 6.8 ± 4.0 vs. 1.8 ± 2.3; and TMD, −2.1 ± 5.7 vs. 5.3 ± 5.2. Overall, scores on the subscales of negative mood state were significantly lower after stimulation using fir essential oil than after the control condition. However, “confusion” increased significantly after stimulation using fir essential oil. The “vigor” subscale score after stimulation using fir essential oil was significantly higher than after the control condition. The total mood disturbance score after stimulation using fir essential oil was significantly lower than after the control condition.

**Figure 4:**
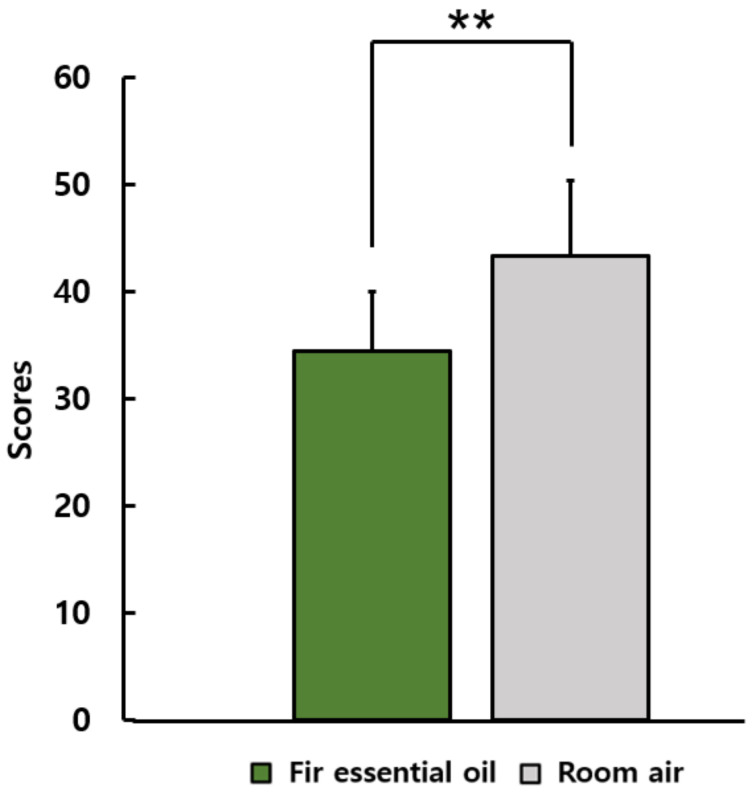
The state anxiety scores of the State-Trait Anxiety Inventory questionnaire after inhaling fir essential oil and room air *N* = 26, mean ± standard deviation, ** *p* < 0.01 by Wilcoxon singed-rank test (one-sided).

Figure 4 shows the results of the STAI. After inhaling fir essential oil, the score on STAI was significantly lower than that after inhaling room air (fir essential oil: 34.4 ± 5.6, room air: 43.3 ± 7.0; *p* < 0.05).

As can be seen from the results of POMS and STAI, negative mood and anxiety decreased, and positive mood increased after inhalation of fir essential oil. These results are consistent with those of previous studies [26,27]. A study on the psychological effects of lavender oil inhalation on female nurses found that stress levels were reduced [26]. Additionally, previous studies have shown that inhalation of cypress oil for four weeks reduced the feeling of depression [27].

### 3.2. Sex Comparison

#### 3.2.1. Physiological Measurement

This study also analyzed sex differences in the effects of inhaling fir essential oil. It is known that olfactory ability differs according to sex [28,29,30]; in general, females have a more sensitive sense of smell [28], an excellent ability to identify odors [29], and a higher olfactory potential than male [30].

**Figure 5:**
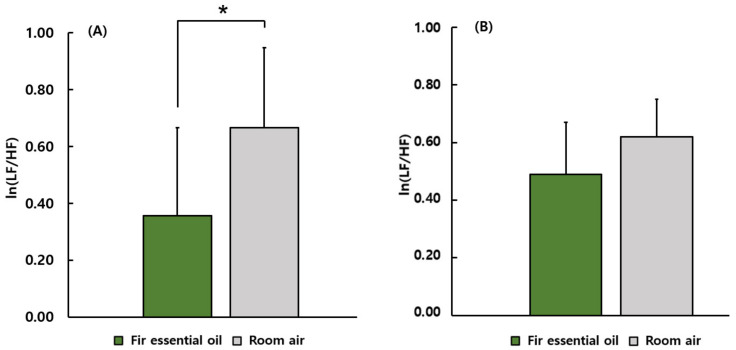
A comparison between females and males in the results of ln(LF/HF). (**A**) The results of the female group. (**B**) The results of the male group. *N* = 11 (the female group), *N* = 9 (the male group), mean ± standard error, * *p* < 0.05 by paired *t*-test (one-sided).

Figure 5 shows a comparison between female and male students in terms of ln(LF/HF) ratio. Among female participants, the ln(LF/HF) ratio during the stimulation using fir essential oil (0.36 ± 0.31) was significantly lower compared with that during the control condition (0.67 ± 0.28). However, no significant differences were found among male participants.

**Figure 6:**
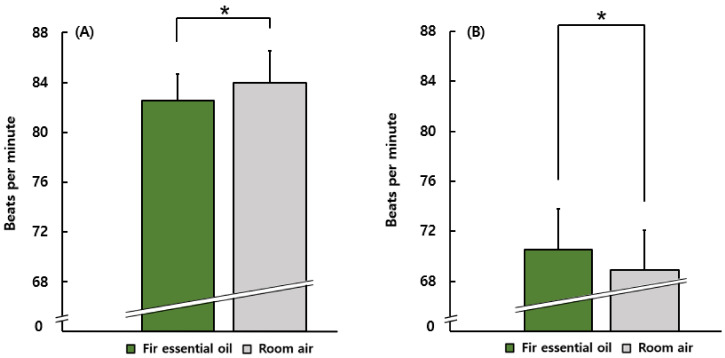
The results of heart rate in comparison between female and male students. (**A**) The results of the female group. (**B**) The results of the male group. *N* = 11 (the female group), *N* = 9 (the male group), mean ± standard error. * *p* < 0.05 by paired *t*-test (one-sided).

Figure 6 shows the results of the comparison between the heart rate of female and male students. Among the female group, the heart rate during stimulation using fir essential oil (82.5 ± 2.2 bpm) was significantly lower than that during the control condition (84.0 ± 2.5 bpm). However, among the male group, the opposite was observed. Their heart rate during olfactory stimulation (70.5 ± 3.3 bpm) was significantly higher than that during the control condition (68.9 ± 3.2 bpm).

#### 3.2.2. Psychological Measurement

**Figure 7:**
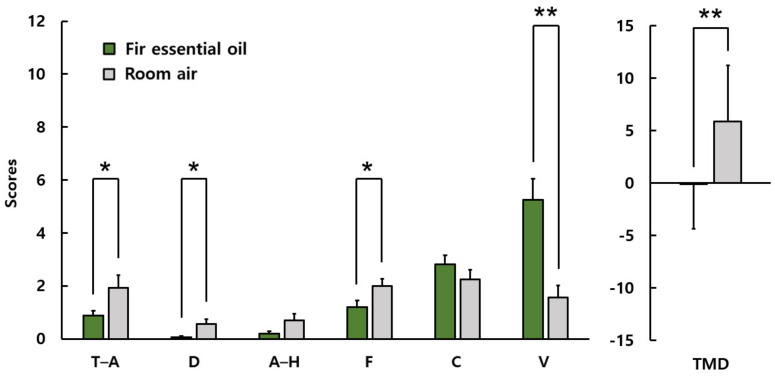
POMS scores of female students. T–A: tension–anxiety; D: depression; A–H: anger–hostility; F: fatigue; C: confusion; V: vigor; TMD: Total Mood Disturbance *N* = 16, mean ± standard deviation, ** *p* < 0.01, * *p* < 0.05 by Wilcoxon singed-rank test (one-sided).

Figure 7 shows the result of POMS for female students. The subscale scores when inhaling fir essential oil and room air were as follows: tension and anxiety, 0.9 ± 1.0 vs. 1.9 ± 2.5; depression, 0.1 ± 0.3 vs. 0.6 ± 1.1; anger–hostility, 0.2 ± 0.5 vs. 0.7 ± 1.5; fatigue, 1.2 ± 1.4 vs. 2.0 ± 1.5; confusion, 2.8 ± 1.8 vs. 2.3 ± 2.1; vigor, 5.3 ± 3.4 vs. 1.6 ± 2.3; and TMD, −0.1 ± 4.2 vs. 5.9 ± 5.3. Negative moods, such as tension–anxiety, depression, and fatigue, were significantly reduced, but no significant differences were found in anger–hostility and confusion. The vigor subscale scores after olfactory stimulation were significantly higher than in the control condition. The total mood disturbance score was significantly lower after olfactory stimulation than in the control condition.

**Figure 8:**
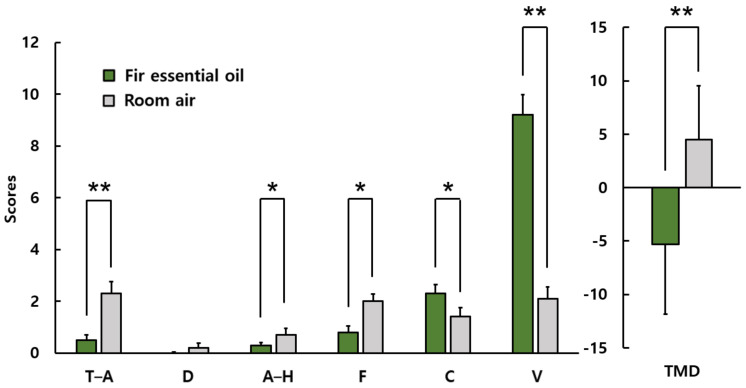
POMS scores of male students. T–A: tension–anxiety; D: depression; A–H: anger–hostility; F: fatigue; C: confusion; V: vigor; TMD: Total Mood Disturbance *N* = 10, mean ± standard deviation, ** *p* < 0.01, * *p* < 0.05 by Wilcoxon singed-rank test (one-sided).

Figure 8 shows the result of POMS in male students. The subscale scores when inhaling fir essential oil and room air were as follows: tension and anxiety, 0.5 ± 1.0 vs. 2.3 ± 2.2; depression, 0.0 ± 0.0 vs. 0.2 ± 0.4; anger–hostility, 0.3 ± 0.5 vs. 0.7 ± 0.9; fatigue, 0.8 ± 1.1 vs. 2.0 ± 1.1; confusion, 2.3 ± 1.6 vs. 1.4 ± 0.8; vigor, 9.2 ± 3.9 vs. 2.1 ± 2.6; and TMD, −5.3 ± 6.5 vs. 4.5 ± 5.0. Negative moods, such as tension–anxiety, anger–hostility, and fatigue were significantly reduced, but no significant differences were found in depression. Vigor subscale scores after olfactory stimulation were significantly higher than in the control condition. The total mood disturbance score was significantly lower after olfactory stimulation than in the control condition. However, unlike the results of female students, confusion increased significantly.

The STAI scores were significantly decreased in both sexes after stimulation using fir essential oil; therefore, no difference was found between male and female students.

Analysis of the differences between male and female participants revealed that heart rate and ln(LF/HF) ratio were significantly decreased when fir essential oil was inhaled among female students. Furthermore, negative mood and anxiety decreased, and positive mood increased. These results are partly consistent with those of all participants. This is consistent with the results of a previous study which studied 13 female university students after inhalation of α-pinene. Their heart rate significantly decreased, parasympathetic nervous activity increased, and subjective feelings of comfort significantly increased [25].

However, among male students, no significant difference was found in sympathetic nervous activity and their heart rate was significantly increased. It is unclear why these results were found in male participants, but it is speculated that the feeling of “confusion” affects the physiological response, such as an increase in heart rate. This is consistent with the results of a previous study [31].

The subjective evaluation of the same scent may differ depending on sex. Female participants showed a positive response to the smell of eugenol, a representative component of vanilla flavor, whereas male participants showed a negative response [31]. It is judged that psychological response may be affected by sex; therefore, additional review will be needed through the accumulation of data in the future.

The importance of this study can be summarized as follows. First, this study was conducted using a forest healing resource of fir trees, which has not been widely used in previous studies. Previous studies have mainly focused on cypress and there is insufficient research on other tree species. Based on the results of this study, it is hoped that fir essential oil can be used in various forest healing activities. Second, short-term inhalation of fir essential oil has a positive effect on physiological and psychological relaxation, even if they do not visit forests. In particular, it can be used for mental and physical relaxation for busy modern people, people with disabilities, the elderly, and people who have difficulty visiting forests. Fir oil inhalation may be very useful in terms of preventive medicine if it is actively used for stress management and mental and physical relaxation in people’s daily lives. Third, the physiological and psychological responses to the inhalation of fir essential oil were analyzed by sex. In previous studies, most experiments were conducted with females and did not demonstrate the difference between males and females. In the case of smell, since there is a difference according to sex, it is necessary to accumulate research data regarding this in the future.

This study has some limitations. First, the generalizability of this study is limited because it was conducted with university students in their 20s. In the future, verification will be required by studying various age groups. Second, it is necessary to diversify physiological indicators. Two physiological indicators were used in this study: HRV and heart rate. However, further research requires objective identification using various physiological indicators, such as cortisol, NK cell activity, and brain activity, for precise evaluation. Third, it is necessary to investigate the physiological and psychological responses to the long-term inhalation of fir essential oil. Although we investigated the responses to inhalation of fir essential oil for a short period of time, no study has clarified the effects of long-term inhalation. Additional research is needed to clarify the physiological and psychological responses with continued inhalation of fir essential oil in daily life. Fourth, additional examinations of more diverse forest healing resources are required. Currently, research on aromatherapy as a forest healing resource has limited the target tree species. In the future, it is hoped that research will investigate the effects of inhalation of essential oils using the representative species of different countries, such as *Pinus densiflora, Pinus koraiensis*, and *Chamaecyparis pisifera* of Korea. Fifth, the number of participants in this study was insufficient for the comparison between females and males. In the future, additional studies will be necessary based on a sufficient number of participants.

## 4. Conclusions

This study revealed the following noteworthy findings regarding the effects of inhalation of fir essential oil on university students: (1) decreased sympathetic nervous activity, (2) improved mood, (3) reduced state anxiety level, and (4) sex differences in physiological and psychological relaxation. In conclusion, it has been found that short-term fir essential oil inhalation induces physiological and psychological relaxation on university students and these responses were different depending on sex.

## Figures and Tables

**Table 1:** Participant information.

	Mean ± Standard Deviation		
Parameter	Total (*n* = 26)	Female (*n* = 16)	Male (*n* = 10)
Age (years)	21.5 ± 1.9	21.2 ± 2.1	22.0 ± 1.4
Height (cm)	166.5 ± 7.5	162.5 ± 5.3	172.9 ± 6.0
Weight (kg)	62.9 ± 11.1	58.4 ± 9.7	70.2 ± 9.3
Body mass index (kg/m^2^)	22.8 ± 0.2	22.1 ± 0.3	23.5 ± 0.2

## Data Availability

The data presented in this study are available on request from the corresponding author.
